# Association between shift work and obesity among female nurses: Korean Nurses’ Survey

**DOI:** 10.1186/1471-2458-13-1204

**Published:** 2013-12-20

**Authors:** Min-Ju Kim, Kuk-Hui Son, Hyun-Young Park, Dong-Ju Choi, Chang-Hwan Yoon, Hea-Young Lee, Eun-Young Cho, Myeong-Chan Cho

**Affiliations:** 1 Division of Cardiovascular and Rare Diseases, Center for Biomedical Science, National Institute of Health, Chungbuk, Korea; 2 Division of Cardiology, Department of Internal Medicine, Seoul National University Bundang Hospital, Bundang, Korea; 3 Korean Nurses Association, Seoul, Korea; 4 Channing Division of Network Medicine, Brigham and Women’s Hospital and Harvard Medical School, Boston, MA, USA; 5 Korea National Institute of Health, Chungbuk, Korea; 6 Department of Internal Medicine, College of Medicine, Chungbuk National University, Chungbuk, Korea

**Keywords:** Shift work, Body mass index, Overweight, Obesity

## Abstract

**Background:**

Shift work has been hypothesized as a risk factor for obesity. In this study, we investigated the association between current shift work and body mass index (BMI) among female nurses in Korea. The relationship between duration of shift work and BMI of the participants was also evaluated.

**Methods:**

This cross-sectional survey evaluated participants in the Korean Nurses’ Survey, conducted from October to December 2011, using web-based self-administered questionnaires. A total of 9,989 nurses were included among 10,000 who registered on the survey web site (5,287 shift workers and 4,702 non-shift workers). Current shift workers were divided into tertiles of shift work duration (0.08–3.00 years, n = 1,732; 3.08–6.75 years, n = 1,731; and 6.83–38.00 years, n = 1,686). The BMI thresholds of overweight and obesity were ≥23 kg/m^2^ and ≥25 kg/m^2^, respectively. Data were analyzed using SPSS software.

**Results:**

Mean participant age was 33.2 ± 8.6 years and the mean BMI was 20.9 ± 2.5 kg/m^2^. There were statistically significant differences in current smoking status, regular drinking habit, dietary habits, regular exercise, sleep problems and self-perceived health status according to duration of shift work. The overall prevalence of overweight/obesity (18.6%) and obesity (7.4%) increased significantly as shift work duration increased from the lowest to highest tertile (*P* for trend <0.001). Multivariate logistic regression analysis revealed no association between current shift work and BMI. However, after adjusting for potential confounders, the participants with the longest duration of shift work were 1.63 (95% CI, 1.22–2.17) times more likely to be overweight or obese than those with the shortest duration. There was a significant positive association between obesity and shift work duration in the unadjusted analysis; however, it was attenuated and no longer significant in the multivariate model.

**Conclusions:**

The duration of shift work was positively associated with prevalence of overweight/obesity in nurses in Korea. Although these findings need to be confirmed in prospective studies, they suggest that special attention should be paid to female nurses with a long duration of shift work.

## Background

Shift work is defined as work outside of daytime hours, including irregular or rotating schedules, and evening and night work [1]. Shift work has been associated with gastrointestinal disease, cardiovascular disease, diabetes, several types of cancer, and metabolic disorders [1-3]. The mechanisms linking shift work to health problems are not clear, but changed circadian rhythm, sleep problems, stress, and lifestyle and behavioral changes such as diet and smoking might be potential mediators [3]. Weight gain in shift workers may be related to several mechanisms which involved alternations of genetic factors, glucose and lipid homeostasis, thermogenic response due to a night eating pattern and neurohumoral factors such as leptin and ghrelin [4].

Obesity is a risk factor for chronic diseases, and a number of studies report that overweight and obesity are more prevalent among shift workers than day workers [5-7]. In addition, several studies show that there are significant correlations between shift work and weight gain, overweight or obesity [8]. Because shift work is an occupational characteristic of nurses, working schedules may influence nurses’ health by increasing the risk for weight gain and obesity [9]. Two studies in nurses working in the United States found that the combined prevalence of overweight and obesity was 55.5% to 65.4% [10,11], similar to or slightly lower than the age-adjusted prevalence among the US general population, which is 63.7% [12].

Shift work is considered a risk factor for obesity, and studies examining this relationship have been conducted in various occupational groups in a number of different countries [8]. However, only a few studies with limited data have reported the impact of occupational characteristics on the body mass index (BMI) of female nurses [8]. In addition, few studies have examined the duration of shift work in relation to obesity [13-15]. Previous founding suggested that the risk of overweight and/or obesity for shift work may increase by at least 39% [15-17]. Therefore, in this study, we investigated the association between current shift work and BMI among female nurses in Korea. The relationship between duration of shift work and the BMI of participants was also evaluated.

## Methods

### Study participants

The Korean Nurses’ Survey is a cross-sectional survey conducted for the purpose of evaluating the association between lifestyle and Korean women’s health. The Korea National Institute of Health collaborated with Korean Nurses Association and Seoul National University’s Bundang Hospital to perform this survey. The Korean Nurses Association sent e-mails containing a direct link to access a web site with an introduction of the survey, questionnaires and an informed consent form. A total of 10,000 nurses registered on the website from October to December 2011 and completed the web-based self-administered questionnaires. Six subjects who were younger than 20 years of age, one who was not employed in nursing, and four with incomplete data on working years and BMI were excluded from the study. A total of 9,989 nurses were included. The nurses were divided into two groups according to current shift work, leaving 5,287 shift workers and 4,702 non-shift workers. Nurses not currently performing shift work were excluded from the main analyses. Among the 5,287 current shift workers, 138 with missing data on shift work duration were excluded; 5,149 were selected for further analysis. The remaining subjects were divided into tertile of shift work duration: Tertile 1, 0.08–3.00 years, n = 1,732; Tertile 2, 3.08–6.75 years, n = 1,731; and Tertile 3, 6.83–38.00 years, n = 1,686. The study protocol was approved by the Institutional Review Board of Seoul National University’s Bundang Hospital. Informed consent was obtained from all study participants.

### Measurements

The questionnaires collected information on demographic characteristics, comorbidities, cardiovascular disease symptoms, medical history, family history of diseases, reproductive health, lifestyle, psychiatric and occupational characteristics, dietary patterns and food intake.

BMI was calculated as body weight (kg) divided by height squared (m^2^). The BMI thresholds were ≥23 kg/m^2^ for overweight and ≥25 kg/m^2^ for obesity. Participants were considered as shift workers if they answered ‘yes’ to the question ‘Do you currently work in shifts?’, and then provided information on shift work duration. Smoking status was characterized as ‘never’, ‘past’, and ‘current’. Current smokers were defined as those who had smoked more than 100 cigarettes in their lifetime and also reported that they were ‘currently smoking’. Participants were considered regular drinkers if they reported consuming alcohol more than once a month. Dietary habits were estimated using the question ‘Do you eat breakfast, lunch and dinner?’ Respondents who answered ‘never’ to eating any of the three meals were characterized as ‘meal skipping’. Subjects were assigned to the regular exercise group if they performed vigorous or moderate-intensity physical activity that caused substantial or slight increase in breathing or heart rate more than once a week. Sleep problems were evaluated by asking about the frequency of difficulty in sleeping or excessive sleep in the past 2 weeks. The four possible responses were: never, 1–6 days, 7–13 days, and every day. Self-perceived health status was determined by asking ‘how you rate your state of health in general’, for which the five responses were: very good, good, fair, so-so, and bad.

### Statistical analysis

Baseline characteristics of the study participants were summarized according to current shift work, and were presented by tertile of shift work duration. Continuous variables were expressed as mean ± standard deviations (SD) and categorical variables as percentages. Statistically significant differences between groups were calculated by the independent *t*-test and one-way analysis of variance (ANOVA) with Duncan’s multiple comparison test. The chi-square test was used to evaluate differences between groups for categorical variables. A linear-by-linear association test was performed to assess trends of overweight and obesity prevalence according to shift work duration. Multivariate logistic regression analysis was conducted to identify associations of shift work with overweight and obesity. Odds ratios (ORs) and 95% confidence intervals (CIs) were calculated. Potential confounding variables that were adjusted for were age (continuous), current smoking status (yes/no), regular drinking habit (yes/no), breakfast skipping (yes/no), regular exercise (yes/no), marital status (married/cohabitation/divorced/widowed/never-married.), family income (less than 30 million won/30–45 million won/45–75 million won/more than 75 million won), education (graduation from 3-year course/graduation from 4-year course/graduate school or higher), sleep problems (never or 1–6 days/7–13 days or every day) and self-perceived health status (very good or good/fair, so-so or bad). Relationships of age, BMI and shift work duration were examined with the Pearson correlation. Spearman correlation coefficients between shift work duration and lifestyle factors were calculated. Adjusted ORs for overweight/obesity and obesity by selected factors were also calculated in a logistic regression model. Values of *P* < 0.05 were considered to be statistically significant. All data were analyzed using SPSS Statistics 21 (SPSS Inc., IBM Corp., Chicago, IL, USA).

## Results

### Baseline characteristics

Baseline characteristics of the study participants according to current shift work are shown in Table 1. The mean age was 33.2 ± 8.6 years and their mean BMI was 20.9 ± 2.5 kg/m^2^. Non-shift workers had a higher mean BMI than current shift workers (21.4 ± 2.6 kg/m^2^ vs. 20.5 ± 2.5 kg/m^2^, *P* < 0.001). Of all subjects, the prevalence of current smokers was relatively low (1.2%) and almost half of the participants were regular drinkers. About 36.5% of subjects skipped breakfast, while 1.3% and 1.0% skipped lunch and dinner, respectively. The prevalence of skipping breakfast was higher in shift workers compared to non-shift workers (43.1% vs. 29.2%). Current shift workers were less likely to exercise regularly than non-shift workers (41.2% vs. 47.3%). Sleep problems for 7–13 days or every day during the past 2 weeks were more frequent among shift workers (27.2%). There were statistically significant differences in self-perceived health status according to current shift work (*P* < 0.001). For further analysis, current shift workers were organized according to tertile of shift work duration. Among the current shift workers, subjects with the longer duration of shift work were more likely to be older (*P* < 0.001) and to have a higher BMI (*P* < 0.001). Those subjects were also more likely to have hypertension, diabetes, hyperlipidemia or breast cancer (*P* = 0.003, *P* < 0.001, *P* = 0.003, *P* < 0.001). The prevalence of regular exercise was the highest in Tertile 2 (44.7%), followed by Tertile 3 (40.1%) and Tertile 1 (38.6%). Difficulty with sleeping or excessive sleep for 7–13 days or every day was most frequent in Tertile 1 (32.9%), followed by Tertile 2 (30.9%) and Tertile 3 (17.8%).

**Table 1:** Mean values of baseline characteristics according to current shift work and tertile of shift work duration

**Variables**	**Total**	**Current shift work**		***P*-value**	**Tertile of shift work duration**			***P*-value**
		**No**	**Yes**		**Tertile 1**	**Tertile 2**	**Tertile 3**	
	**(n = 9,989)**	**(n = 4,702)**	**(n = 5,287)**		**(n = 1,732)**	**(n = 1,731)**	**(n = 1,686)**	
Age (years)	33.2 ± 8.6	37.6 ± 9.2	29.2 ± 5.6	<0.001	24.9 ± 2.9^c^	28.1 ± 3.1^b^	34.7 ± 5.1^a^	<0.001
BMI (kg/m^2^)	20.9 ± 2.5	21.4 ± 2.6	20.5 ± 2.5	<0.001	20.0 ± 2.1^c^	20.2 ± 2.4^b^	21.3 ± 2.7^a^	<0.001
Hypertension	220 (2.2)	193 (4.1)	27 (0.5)	<0.001	3 (0.2)	6 (0.3)	16 (0.9)	0.003
Diabetes	43 (0.4)	31 (0.7)	12 (0.2)	0.001	3 (0.2)	4 (0.2)	5 (0.3)	<0.001
Osteoporosis	102 (1.0)	79 (1.7)	23 (0.4)	<0.001	1 (0.1)	6 (0.3)	13 (0.8)	0.003
Hyperlipidemia	57 (7.6)	472 (10.0)	285 (5.4)	<0.001	40 (2.3)	78 (4.5)	165 (9.8)	<0.001
Breast cancer	87 (0.9)	73 (1.6)	14 (0.3)	<0.001	1 (0.1)	3 (0.2)	10 (0.6)	0.007
Current smoking status				0.335				<0.001
No	9874 (98.8)	4653 (99.0)	5221 (98.8)		1697 (98.0)	1718 (99.2)	1675 (99.3)	
Yes	115 (1.2)	49 (1.0)	66 (1.2)		35 (2.0)	13 (0.8)	11 (0.7)	
Regular drinking habit				<0.001				<0.001
No	5144 (51.5)	2683 (57.1)	2461 (46.5)		625 (36.1)	788 (45.5)	993 (58.9)	
Yes	4845 (48.5)	2019 (42.9)	2826 (53.5)		1107 (63.9)	943 (54.5)	693 (41.1)	
Marital status				<0.001				<0.001
Married (remarriage)	4589 (45.9)	3087 (65.7)	1502 (28.4)		88 (5.1)	344 (19.9)	1032 (61.2)	
Cohabitation	16 (0.2)	6 (0.1)	10 (0.2)		3 (0.2)	2 (0.1)	4 (0.2)	
Divorced	246 (2.5)	127 (2.7)	119 (2.3)		31 (1.8)	42 (2.5)	40 (2.4)	
Widowed	36 (0.4)	34 (0.7)	2 (0.0)		0 (0.0)	0 (0.0)	2 (0.1)	
Never-married	5102 (51.1)	1448 (30.8)	3654 (69.1)		1610 (93.0)	1341 (77.5)	608 (36.1)	
Family income				<0.001				<0.001
Less than 30 million won	1259 (12.6)	490 (10.4)	769 (14.5)		401 (23.2)	247 (14.3)	96 (5.7)	
30-45 million won	2787 (27.9)	1002 (21.3)	1785 (33.8)		605 (34.9)	685 (39.6)	454 (26.9)	
45-75 million won	3545 (35.5)	1802 (38.3)	1743 (33.0)		472 (27.3)	522 (30.2)	699 (41.5)	
More than 75 million won	2389 (24.0)	1408 (29.9)	990 (18.7)		254 (14.7)	277 (16.0)	437 (25.9)	
Education				<0.001				<0.001
Graduation from three-year course	3469 (34.7)	1294 (27.5)	2175 (41.1)		845 (48.8)	733 (42.3)	528 (31.3)	
Graduation from four-year course	4839 (48.4)	2083 (44.3)	2756 (52.1)		880 (50.8)	954 (55.1)	862 (51.1)	
Graduate school or higher	1681 (16.8)	1325 (28.2)	356 (6.7)		7 (0.4)	44 (2.5)	296 (17.6)	
Dietary habits								
Breakfast skipping	3649 (36.5)	1371 (29.2)	2278 (43.1)	<0.001	749 (43.2)	815 (47.1)	647 (38.4)	<0.001
Lunch skipping	129 (1.3)	23 (0.5)	106 (2.0)	<0.001	50 (2.9)	35 (2.0)	19 (1.1)	<0.001
Dinner skipping	96 (1.0)	38 (0.8)	58 (1.1)	0.140	20 (1.2)	23 (1.3)	14 (0.8)	<0.001
Regular exercise				<0.001				<0.001
No	5588 (55.9)	2478 (52.7)	3110 (58.8)		1063 (61.4)	957 (55.3)	1010 (59.9)	
Yes	4401 (44.1)	2224 (47.3)	2177 (41.2)		669 (38.6)	774 (44.7)	676 (40.1)	
Sleep problems, during the last 2 weeks				<0.001				<0.001
Never	4540 (45.4)	2815 (59.9)	1725 (32.6)		477 (27.5)	494 (28.5)	707 (41.9)	
1-6 days	3538 (35.4)	1414 (30.1)	2124 (40.2)		685 (39.5)	702 (40.6)	679 (40.3)	
7-13 days	1187 (11.9)	304 (6.5)	883 (16.7)		333 (19.2)	331 (19.1)	202 (12.0)	
Every day	724 (7.2)	169 (3.6)	555 (10.5)		237 (13.7)	204 (11.8)	98 (5.8)	
Self-perceived health status				<0.001				0.003
Very good	716 (7.2)	389 (8.3)	327 (6.2)		119 (6.9)	105 (6.1)	92 (5.5)	
Good	4545 (45.5)	2219 (47.2)	2326 (44.0)		804 (46.4)	756 (43.7)	710 (42.1)	
Fair	3837 (38.4)	1735 (36.9)	2102 (39.8)		662 (38.2)	666 (38.5)	716 (42.5)	
So-so	753 (7.5)	290 (6.2)	463 (8.8)		134 (7.7)	174 (10.1)	143 (8.5)	
Bad	138 (1.4)	69 (1.5)	69 (1.3)		13 (0.8)	30 (1.7)	25 (1.5)	

Data are expressed as mean ± SD or percentage. Statistical differences between groups were compared with independent sample *t*-test and one-way ANOVA for continuous variables and chi-square test for categorical variables.

^a, b, c^The same letters indicate non-significant difference between groups based on Duncan’s multiple comparison test.

### Prevalence of overweight and obesity

Table 2 shows the prevalence of overweight and obesity according to current shift work and shift work duration. The overall prevalence of overweight or obesity and obesity was 18.6% and 7.4%, respectively. Non-shift workers had a higher prevalence of overweight and obesity than shift workers (*P* < 0.001). The prevalence increased significantly as shift work duration increased from Tertile 1 to Tertile 3 (*P* for trend <0.001).

**Table 2:** Prevalence of overweight/obesity and obesity according to current shift work and tertile of shift work duration

**Variables**	**Total**		**Current shift work**				***P*-value**	**Tertile of shift work duration**						***P *for trend**
			**No**		**Yes**			**Tertile 1**		**Tertile 2**		**Tertile 3**		
	**(n = 9,989)**		**(n = 4,702)**		**(n = 5,287)**			**(n = 1,732)**		**(n = 1,731)**		**(n = 1,686)**		
BMI category														
< 23 kg/m^2^	8134	(81.4)	3578	(76.1)	4556	(86.2)	<0.001	1593	(92.0)	1546	(89.3)	1300	(77.1)	<0.001
≥ 23 kg/m^2 a^	1855	(18.6)	1124	(23.9)	731	(13.8)		139	(8.0)	185	(10.7)	386	(22.9)	
BMI category														
< 25 kg/m^2^	9253	(92.6)	4274	(90.9)	4979	(94.2)	<0.001	1678	(96.9)	1647	(95.1)	1524	(90.4)	<0.001
≥ 25 kg/m^2 b^	736	(7.4)	428	(9.1)	308	(5.8)		54	(3.1)	84	(4.9)	162	(9.6)	

Data are expressed as n (%) and tested by chi-square test.

^a^Overweight and obesity combined.

^b^Obesity.

### Results of multivariate logistic regression analysis of overweight and obesity

The results of multivariate logistic regression analysis of overweight and obesity according to current shift work are presented in Additional file 1. The ORs of overweight/obesity and obesity in unadjusted analysis were 0.51 (95% CI, 0.46–0.57) and 0.62 (95% CI, 0.53–0.72), respectively. However, no association was found in any of the adjusted models.

When the non-shift work group was used as a reference, Tertile 3 of shift work duration had a significantly increased risk for overweight/obesity (OR, 1.24; 95% CI, 1.07-1.43) and obesity (OR, 1.32; 95% CI, 1.08-1.62) after fully adjusting for covariates, including age [see Additional file 2]. Unadjusted, age-adjusted, and multivariate-adjusted ORs for overweight and obesity according to duration of shift work are shown in Table 3. When the overweight and obese participants were combined into one group in the unadjusted analysis, the ORs of overweight/obesity in Tertile 3 of shift work duration was 3.40 (95% CI, 2.77-4.19) compared with Tertile 1 (*P* < 0.001). After adjusting for potential confounding variables in multivariate logistic regression analysis, the participants in Tertile 3 were 1.63 (95% CI, 1.22-2.17) times more likely to be overweight/obese than those in Tertile 1 (*P* = 0.001). Similar results were also observed in overweight subjects. The participants in Tertile 3 were significantly associated with the increased risk of overweight (data not shown). There were significant, positive correlations between obesity and shift work duration in the unadjusted analysis. Although the association was statistically not significant after adjustment for age, statistically significant correlations were observed after adjustment for current smoking status, regular drinking habit, breakfast skipping and regular exercise (*P* = 0.028). However, no association was observed in the multivariate model. Tertile 2 showed the increased trends for an increased risk of overweight/obesity or obesity in the adjusted models, although this association was not statistically significant. Age, current smoking status, marital status, family income, education, breakfast skipping, regular exercise and sleep problems were each significantly associated with overweight/obesity when included as covariates in the logistic regression models [see Additional file 3]. The factors that were significantly associated with obesity included age, current smoking status, marital status, breakfast skipping, regular exercise and self-perceived health status [see Additional file 4].

**Table 3:** Multivariate-adjusted odds ratios (ORs) for overweight/obesity and obesity according to tertile of shift work duration

		**Unadjusted**			**Model 1**			**Model 2**			**Model 3**		
		**OR(95% CI)**		***P*-value**	**OR(95% CI)**		***P*-value**	**OR(95% CI)**		***P*-value**	**OR(95% CI)**		***P*-value**
BMI <23 kg/m^2^ vs. BMI ≥23 kg/m^2^	Shift work duration												
	Tertile 1	1	(reference)		1	(reference)		1	(reference)		1	(reference)	
	Tertile 2	1.37	(1.09-1.73)^**^	0.007	1.09	(0.86-1.39)	0.471	1.13	(0.89-1.44)	0.311	1.11	(0.87-1.42)	0.404
	Tertiel 3	3.40	(2.77-4.19)^***^	<0.001	1.67	(1.28-2.19)^***^	<0.001	1.79	(1.37-2.35)^***^	<0.001	1.63	(1.22-2.17)^**^	0.001
BMI <25 kg/m^2^ vs. BMI ≥25 kg/m^2^	Shift work duration												
	Tertile 1	1	(reference)		1	(reference)		1	(reference)		1	(reference)	
	Tertile 2	1.59	(1.12-2.25)^*^	0.010	1.23	(0.86-1.76)	0.256	1.26	(0.88-1.81)	0.206	1.21	(0.84-1.74)	0.315
	Tertile 3	3.30	(2.41-4.53)^***^	<0.001	1.48	(1.00-2.18)	0.050	1.56	(1.05-2.31)^*^	0.028	1.37	(0.91-2.09)	0.135

CI, confidence interval.

Model 1: adjusted for age

Model 2: adjusted for age, current smoking status, regular drinking habit, breakfast skipping and regular exercise.

Model 3: adjusted for age, current smoking status, regular drinking habit, breakfast skipping, regular exercise, marital status, family income, education, sleep problem and self-perceived health status.

^*^P < 0.05, ^**^P < 0.01, ^***^P < 0.001.

## Discussion

The purpose of this study was to examine the relationship between shift work and BMI among nurses in Korea. The prevalence of overweight/obesity and obesity significantly increased with increasing shift work duration. Although there was no association between current shift work and BMI, we found that the subjects in the highest tertile of shift work duration were more likely to be overweight/obese after adjustment for potential confounders.

In this sample of 9,989 female nurses, 66.5% were of normal weight, suggesting that overweight or obesity is not common in the population of nurses in Korea. In addition, 18.6% of the participants were either overweight (11.2%) or obese (7.4%), which is lower than the age-standardized obesity prevalence of 25.7% in the general Korean population, as reported by the Korean National Health and Nutrition Examination Survey (KNHANES) [18]. These results might be explained either by a healthy-worker effect among nurses or the large proportion of participants who were 20–29 years of age (44.4%) and 30–39 years of age (33.8%). The BMI in non-shift workers was higher than that in current shift workers, which might have been influenced by the relatively older age of non-shift workers, as well as other potential confounding factors. When adjusted for potential confounders in multivariate logistic regression analysis, no significant association was found between current shift work and BMI. Because non-shift workers had not provided any information about past shift work experience, they were excluded from the main analyses.

In a further analysis, we also found that most of the subjects in the lowest tertile of shift work duration had never married (93.0%), whereas those in the highest tertile were more likely to be married (61.2%). As shown in Additional files 3 and 4, married women who were current shift workers had a higher risk of overweight/obesity and obesity compared to never-married women, which is consistent with an earlier study [16]. In the present study, the finding that nurses with lower levels of education and income had an increased risk of overweight/obesity could be explained by a decreased interest in health problems in this group.

Several studies report finding associations between shift work and weight gain, overweight or obesity [14-16,19]. The female workers in electronic device factories in Malaysia who worked night shifts had significantly elevated ORs for being overweight, even after adjustments [16]. A study including 377 shift workers and non-shift workers found a positive relationship between duration of shift work and BMI [14], and a cross-sectional survey found that longer exposure to shift work was a highly significant predictor of increased BMI [15]. Our results were consistent with those obtained in earlier studies; multiple logistic regression revealed that Tertile 3 of shift work duration had a significantly increased risk for overweight/obesity (OR, 1.24; 95% CI, 1.07-1.43) and obesity (OR, 1.32; 95% CI, 1.08-1.62) compared with non-shift work group. When the shortest duration of shift work was used as a reference, the nurses with the longest duration of shift work had a 1.63 times greater risk of overweight/obesity. Similarly, a cross-sectional study from Australia found that nurses working shifts were 1.15 times more likely to be overweight or obese than day workers, and night-only shift work was associated with obesity only [20,21]. A study in 85 hospital shift workers reported that mean weight gain was greater in those on evening and night shifts (4.3 kg) than in those on day shifts (0.9 kg) [13]. Although our findings showed no association between shift work duration and obesity in the multivariate model, statistically significant correlations was observed after adjustment for age, current smoking status, regular drinking habit, breakfast skipping and regular exercise. This might be explained by the relatively low proportion of obese nurses among current shift workers (5.8% total; 3.1% in Tertile 1, 4.9% in Tertile 2, and 9.6% in Tertile 3). Therefore, we combined overweight and obesity into one category as dependent variables. For further analysis, we selected the participants over the age of 30 years. Tertile 2 and Tertile 3 showed the significant association with overweight/obesity after adjustment for potential confounders (OR, 1.45; 95% CI, 1.07-1.98 and OR, 1.45; 95% CI, 1.04-2.02). There was a significant association between obesity and Tertile 3 in the unadjusted analysis (OR, 1.59; 95% CI, 1.07-2.36); however, it was no longer significant in the adjusted models.

It is well known that lifestyle factors are potential mediators linking shift work to BMI [3]. In a logistic regression model including confounding factors as covariates, the factors that remained significantly associated with overweight/obesity were age, current smoking status, marital status, family income, education, breakfast skipping and sleep problems. It is known that inadequate sleep and poor quality sleep are associated with obesity [7]. Consistent with those findings, our results showed that sleep problems were associated with overweight/obesity [see Additional file 3]. Approximately 27% of shift workers responded that they had difficulty with sleeping or excessive sleep for 7–13 days or every day during the past 2 weeks. Interestingly, sleep problems were reported most frequently in Tertile 1 (32.9%), followed by Tertile 2 (30.9%) and Tertile 3 (17.8%), showing a higher prevalence among subjects who started shift work recently. One previous study reported that nurses’ diet and exercise habits were negatively influenced by sleep disturbance by working at night [22], and that they also faced changes in eating habits and food selection. Previous studies found that midnight shift workers had the highest total energy intake and usually consumed more calories in the evening [4]. In another study, 40% of nurses who recognized that they were overweight or obese responded that they ate a healthy diet and exercise regularly, but could not lose excess weight [23]. In the present study, 43.1% of shift workers skipped breakfast. The percentage was highest in Tertile 2 (47.1%), followed by Tertile 1 (43.2%), and Tertile 3 (38.4%). In addition, there was an inverse relationship with breakfast skipping and the risk of overweight and obesity. In the current study, we did not investigate diet quality, types, or amount of food eaten and energy intake; this should be further examined. In general, physical activity is inversely associated with obesity; however, our results indicated that regular exercise had a positive relationship with overweight/obesity. The prevalence of shift workers who exercised regularly was 40.3% in those with BMI <23 kg/m^2^ and 46.2% in those with BMI ≥23 kg/m^2^. This might imply that nurses who recognized that they were overweight or obese were more likely to exercise regularly. Because this was a cross-sectional study, additional investigations are needed for better understanding of the relationship between shift work and physical activity. Interestingly, the participants with the longest duration of shift work had a significantly increased risk of hyperlipidemia after adjustment for age (OR, 1.68; 95% CI, 1.11-2.55).

This study included a large, representative sample of nurses in Korea and it confirmed an association between increasing duration of shift work and overweight/obesity after considering obesity-related lifestyle factors as confounders. However, there are some study limitations. First, as the study was cross-sectional in design, it is difficult to conclude that the relationship between shift work and BMI was causal. Further longitudinal study will be needed to achieve higher levels of evidence. Second, our data were collected from self-reported questionnaires. Additional metabolic markers should be included in future research for a better understanding of the relationship between shift work and body weight. Third, nurses not currently performing shift work might be misclassified because they had not provided any information about past shift work experience. Finally, shift work exposures at multiple aspects, such as frequency of night shifts, duration of each shifts, speed and direction of shift rotation, were not collected in the current study. Accordingly, further studies will be needed to assess the relationships of different kinds of schedules and duration of shift work to obesity.

## Conclusions

In conclusion, we found that increased duration of shift work was associated with higher risk of overweight/obesity in shift-working nurses in Korea. Although our findings need to be confirmed in prospective studies, they suggest that special attention should be paid to female nurses with long durations of shift work. It would also be valuable to investigate which shift schedules are related to obesity.

## Abbreviations

BMI: Body mass index; SD: Standard deviations; ANOVA: One-way analysis of variance; ORs: Odds ratios; CIs: Confidence intervals; KNHANES: Korean National Health and Nutrition Examination Survey.

## Competing interests

The authors declare that they have no competing interests.

## Authors’ contributions

MJK performed the statistical analysis and participated in drafting the manuscript. KHS contributed data collection and management. HYP participated in the design of the study, revised the manuscript for important intellectual content, and gave final approval of the version to be published. DJC is the principal investigator of this study, and made substantial contributions to study design and discussion. CHY and HYL collected data, and contributed to study design and discussion. EYC contributed data analysis and involved in revising the manuscript. MCC is the general supervision of the research group, and made substantial contributions to design of the study. All authors read and approved the final manuscript.

## Pre-publication history

The pre-publication history for this paper can be accessed here:

http://www.biomedcentral.com/1471-2458/13/1204/prepub

## Supplementary Material

Additional file 1 Multivariate-adjusted odds ratios (ORs) for overweight/obesity and obesity according to current shift work. Click here for file

Additional file 2 Multivariate-adjusted odds ratios (ORs) for overweight/obesity and obesity according to shift work duration. Click here for file

Additional file 3 Adjusted odds ratios (ORs) for overweight/obesity by selected factors among current shift workers. Click here for file

Additional file 4 Adjusted odds ratios (ORs) for obesity by selected factors among current shift workers. Click here for file
